# Frailty, periinterventional complications and outcome in patients undergoing percutaneous mitral and tricuspid valve repair

**DOI:** 10.1007/s00392-024-02397-3

**Published:** 2024-02-15

**Authors:** Matthieu Schäfer, Hannah Nöth, Clemens Metze, Christos Iliadis, Maria Isabel Körber, Marcel Halbach, Stephan Baldus, Roman Pfister

**Affiliations:** University of Cologne, Faculty of Medicine and University Hospital Cologne, Clinic III for Internal Medicine, Köln, Germany

**Keywords:** Frailty, Bleeding, MitraClip, Infection, Acute Kidney Injury

## Abstract

**Background:**

Frailty is common in elderly and multimorbid patients and associated with increased vulnerability to stressors.

**Methods:**

In a single centre study frailty according to Fried criteria was assessed in consecutive patients before transcatheter mitral and tricuspid valve repair. Postprocedural infections, blood transfusion and bleeding and renal failure were retrospectively assessed from records. Median follow-up time for survival was 560 days (IQR: 363 to 730 days).

**Results:**

90% of 626 patients underwent mitral valve repair, 5% tricuspid valve repair, and 5% simultaneous mitral and tricuspid valve repair. 47% were classified as frail. Frailty was associated with a significantly increased frequency of bleeding (16 vs 10%; *p* = 0.016), blood transfusions (9 vs 3%; *p* =  < 0.001) and infections (18 vs 10%; *p* = 0.006), but not with acute kidney injury (20 vs 20%; *p* = 1.00). Bleeding and infections were associated with longer hospital stays, with a more pronounced effect in frail patients (interaction test *p* < 0.05, additional 3.2 and 4.1 days in frail patients, respectively). Adjustment for the occurrence of complications did not attenuate the increased risk of mortality associated with frailty (HR 2.24 [95% CI 1.62–3.10]; *p* < 0.001).

**Conclusions:**

Bleeding complications and infections were more frequent in frail patients undergoing transcatheter mitral and tricuspid valve repair and partly explained the longer hospital stay. Albeit some of the complications were associated with higher long-term mortality, this did not explain the strong association between frailty and mortality. Further research is warranted to explore interventions targeting periprocedural complications to improve outcomes in this vulnerable population.

**Graphical abstract:**

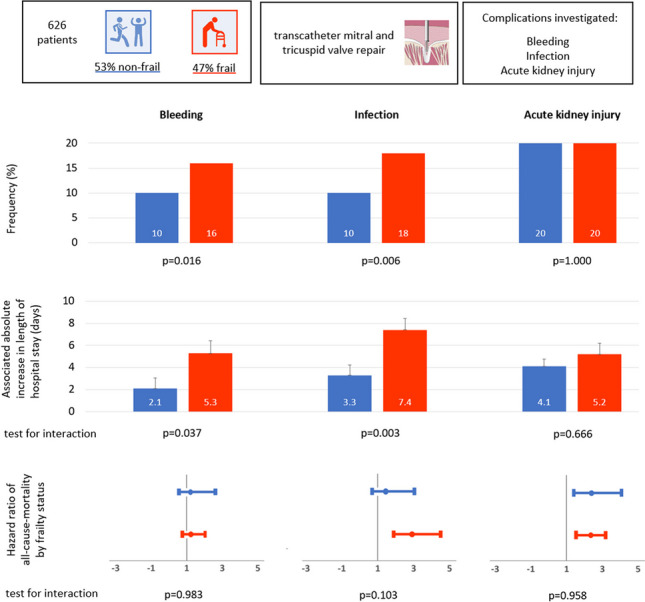

**Supplementary information:**

The online version contains supplementary material available at 10.1007/s00392-024-02397-3.

## Introduction

Catheter-based percutaneous heart valve repair has become an established treatment option for patients with symptomatic mitral and tricuspid valve regurgitation who are considered inoperable or at high surgical risk by the heart team. The catheter-based procedure is generally safe and less stressful for the patient than cardiac surgery. In addition to severe cardiac and extracardiac morbidity, frailty is a widely accepted criterion indicating increased surgical risk and an argument against surgery and in favour of transcatheter treatment [1]. As a result, frailty is common in patients undergoing percutaneous heart valve treatment.

Frailty is a complex clinical condition characterised by an increased vulnerability to internal and external stressors, resulting in an increased risk of adverse health outcomes particularly in the context of medical interventions [2]. In the general population and in patients with cardiovascular disease, frailty is a strong predictor of mortality. Furthermore, frailty is associated with mortality, slower postoperative recovery and longer hospital stay after cardiac surgery and transcatheter aortic valve repair [3, 4]. In a pilot study on the impact of frailty on outcomes in patients undergoing percutaneous mitral valve repair, we showed that approximately one out of two patients was frail and frailty was associated with longer hospital stay and higher mid-term mortality [5]. The underlying reasons for these associations are unknown but are of great clinical interest as they may provide potential targets for future interventions. We hypothesised that periinterventional complications may be more common in frail patients undergoing TMTVR and may be particularly relevant to clinical outcomes in this vulnerable population.

In a large monocentric retrospective analysis, we examined the frequency of post-procedural complications such as infection, bleeding and acute kidney injury (AKI) by frailty status and their impact on subsequent patient outcomes.

## Methods

### Study design and patient population

We conducted a retrospective analysis using our single centre database. All consecutive patients who underwent TMTVR for valve regurgitation at the Heart Centre of the University Hospital of Cologne, Germany, between May 2014 and December 2019 were eligible for inclusion in our registry. Patients who refused consent or had significant language barrier were excluded. All patients had an indication for treatment of mitral or tricuspid regurgitation according to current guidelines at the time [6, 7] and were discussed in a multidisciplinary heart team. Only patients at high or prohibitive surgical risk and amenable to the appropriate catheter techniques were treated. Valve repair with Cardioband, Pascal (both Edwards Lifesciences), MitraClip or TriClip (both Abbott Vascular) was performed as previously described [8–11]. Patients with acute cardiac decompensation were excluded from the study.

Baseline demographic and clinical characteristics were obtained from medical records or the automated information system (ORBIS, Agfa Healthcare, Bonn, Germany).

### Frailty-assessment

Frailty was assessed on the day before the intervention according to the criteria defined by Fried et al. as previously reported [5]. Five components of the frailty syndrome were measured, and 1 point was awarded for each criterion met to specification. The five components were: 1) unintentional weight loss of > 5% of body weight or > 4.5 kg in the past year; 2) weakness: grip strength of the dominant hand measured with a Jamar dynamometer and averaged from 3 measurements < 18kg in women and < 30kg in men; 3) self-reported exhaustion: “In the past week (during 3 days or more) I felt that everything I did was an effort and/or I was unable to do anything”; 4) slowness: walking time for a separate 4.57-m walk test > 6s; and 5) low physical activity, derived from 1 question for practical reasons as a modification to the original Fried score which used a physical activity questionnaire: "In the past month (4 weeks), has your heart failure prevented you from doing the things you want to do by making you sit or lie down to rest during the day?", answered as 4 or 5 (on a 6-point Likert scale from 0 = not at all to 5 = very much). This may be a reasonable approximation because objectively measured activity levels in patients with heart failure show a high correlation with symptom burden–based questionnaires such as the Minnesota living with heart failure questionnaire. Patients who met at least 3 of the 5 criteria were classified as frail.

### Endpoints

Procedural effect was assessed by transthoracic echocardiography before discharge, and valve regurgitation was graded according to current guidelines [6, 12]. Efficacy and safety endpoints were assessed retrospectively from records and defined according to the Mitral Valve Academic Research Consortium (MVARC) criteria [13] unless otherwise stated. All 3 stages of AKI based on the MVARC criteria were considered and combined into AKI of any stage. Procedural success was defined as technical success and reduction of regurgitation to grade ≤ 2. Clinically manifest infection was assessed retrospectively from the medical record or discharge letter. Only infections starting within 72 h after the procedure were included to avoid reverse causality in the association with length of hospital stay. The type of infection such as pneumonia and urinary tract infection was also assessed.

In addition to bleeding according to MVARC criteria, bleeding requiring blood transfusion was assessed. Predefined sites of bleeding were the access site, the jugular central venous catheter, the gastrointestinal tract and the urological tract.

Vital status was assessed in March 2021. If a patient’s death was not already documented in our automated information system, patients or their general practitioner were contacted by telephone.

### Statistical analysis

Frail patients were compared with non-frail patients with respect to baseline characteristics, periprocedural complications and clinical course including length of hospital stay and long-term mortality. All continuous variables were not normally distributed and are presented as median with interquartile range. The Mann-Whitney U test was used to calculate the significance of differences by subgroup. Nominal and ordinal data are expressed as numbers and percentages and the significance of differences was calculated using the χ2 test. If the expected value in any cell was < 5, the Fisher exact test was used. Associations of complications with length of hospital stay were analysed using univariable and multivariable linear regression analysis. Survival curves were estimated using the Kaplan-Meier method and compared using log-rank test. Cox proportional hazards models were used to examine the effect of individual characteristics on outcome. Observations were censored at 2 years, at the date of death or last confirmed alive status, whichever came first. All tests were 2-tailed, and a *p* < 0.05 was considered statistically significant. Statistical analyses were performed using SPSS Statistics, version 29 (IBM Corp, Armonk, NY).

## Results

### Baseline characteristics

Six hundred and seventy-nine patients were admitted for percutaneous mitral or tricuspid valve repair at our institution during the study period. Thirty-three patients were excluded because they refused to participate (*n* = 16), were unable to give consent due to critical illness in intensive care (*n* = 11) or had a significant language barrier (*n* = 6). Twenty patients were excluded because their frailty status could not be classified due to missing data on individual frailty criteria. Finally, 626 patients were included in the analysis. The median age was 79 (IQR 73–83) years and 56% of patients were male.

Two hundred and ninety-two patients (47%) were classified as frail according to Fried criteria. Frail patients were significantly older and had significantly higher EuroScore II and NT-proBNP levels. Renal function was worse and baseline haemoglobin was significantly lower. Frail patients were significantly more likely to have atrial fibrillation and to live in a nursing home. Frail patients were also significantly more likely to be in NYHA functional class III or IV (Table 1).

**Table 1:** Baseline patient characteristics in the total population and by frailty status

	Data available	Overall (*n* = 626)	Non-frail (*N* = 334)	Frail (*N* = 292)	*p*-value
Age, yrs	626	79 (73–83)	78 (72–83)	80 (74–84)	0.007
Male	626	349 (56)	196 (59)	153 (52)	0.126
Body mass index, kg/m^2^	621	24.7 (22.6–28.4)	24.6 (22.7–28.0)	25.2 (22.0–28.7)	0.818
Living in nursing home	586	13 (2)	3 (1)	10 (4)	0.046
EuroScore II, %	613	5.4 (3.1–5.4)	5.1 (2.8–9.1)	5.8 (3.4–11.0)	0.008
NYHA functional class III-IV	619	545 (88)	271 (82)	274 (95)	< 0.001
LVEF	545				0.333
> 50%		248 (46)	124 (43)	124 (50)	
30%-50%		173 (32)	96 (33)	77 (30)	
< 30%		124 (23)	71 (24)	53 (21)	
NT-proBNP, ng/l	500	2628 (1412–5990)	2235 (1276–4878)	3258 (1628–7378)	< 0.001
Estimated glomerular filtration rate (CKD-EPI), ml/min	603	45 (33–62)	47 (36–65)	40 (28–57)	< 0.001
Chronic kidney disease	608	454 (74)	229 (70.2)	225 (80)	0.009
Dialysis	605	16 (3)	3 (1)	13 (5)	0.005
Haemoglobin, g/dl	625	12.3 (10.8–13.4)	12.5 (11.2–13.6)	12.1 (10.6–13.2)	< 0.001
Diabetes mellitus	625	157 (25)	81 (24)	76 (26)	0.644
Hypertension	623	465 (75)	247 (74(	218 (75)	0.783
Chronic obstructive pulmonary disease	623	89 (14)	44 (13)	45 (16)	0.491
Peripheral artery disease	623	74 (12)	33 (10)	41 (14)	0.108
Coronary artery disease	578	333 (58)	175 (57)	158 (58)	0.800
Prior myocardial infarction	604	151 (25)	77 (24)	74 (26)	0.572
Atrial fibrillation	626	427 (68)	213 (64)	214 (73)	0.013
Previous cardiac surgery	612	208 (34)	120 (37)	88 (31)	0.104
Cause of MV regurgitation	551				0.401
Functional		292 (53)	164 (55)	128 (50)	
Degenerative		224 (41)	113 (38)	111 (44)	
Combined		35 (6)	20 (7)	15 (6)	
Cause of TV regurgitation	55				1.000
Functional		55 (100)	24 (100)	31 (100)	
Degenerative		0	0	0	
Combined		0	0	0	
Baseline regurgitation of treated valves					
MV	548				0.069
Grade 3		213 (39)	125 (42)	88 (35)	
Grade 4		335 (61)	170 (58)	165 (65)	
TV	49				0.869
Grade 3		25 (51)	11 (52)	14 (50)	
Grade 4		24 (49)	10 (48)	14 (50)	

*CKD-EPI* = Chronic Kidney Disease Epidemiology Collaboration, *LVEF* = left ventricular ejection fraction, *MV* = Mitral Valve, *NT-proBNP* = N-terminal prohormone of brain natriuretic peptide, *NYHA* = New York Heart Association, *TV* = Tricuspid Valve

### Procedure details

Four hundred and eighty-four patients (77%) underwent edge-to-edge mitral valve repair with MitraClip and 39 (6%) with PASCAL. Eleven patients (2%) underwent edge-to-edge tricuspid valve repair, and 31 patients (5%) underwent simultaneous edge-to-edge repair of both valves. Twenty-six patients (4%) underwent direct annuloplasty of the mitral valve and 16 patients (3%) of the tricuspid valve (Cardioband). In 19 of 626 patients (3%) the device could not be implanted for technical, procedural or morphological reasons. Devices used and technical success were similar in frail and non-frail patients (Table 2).

**Table 2:** Procedural details in the total population and by frailty status

	Data available	Overall (*n* = 626)	Non-frail (*N* = 334)	Frail (*N* = 292)	*p*-value
Intervention	626				
MV Clip		484 (77)	255 (76)	229 (78)	
TV Clip		11 (2)	8 (2)	3 (1)	
MV + TV Clip		31 (5)	11 (3)	20 (7)	
Cardioband MV		26 (4)	15 (5)	11 (4)	
Cardioband TV		16 (3)	7 (2)	9 (3)	
PASCAL MV		39 (6)	29 (9)	10 (3)	
No implantation		19 (3)	9 (3)	10 (3)	
Reduction of regurgitation to ≤ grade 2 at follow-up	481	423 (88)	242 (87)	181 (89)	0.571
Bleeding complication (MVARC)	603				
any		76 (12)	31 (10)	45 (16)	0.016
Minor		59 (9)	22 (7)	37 (13)	
Major		13 (2)	7 (2)	6 (2)	
Extensive		3 (1)	1 (0)	2 (1)	
Life threatening		1 (0)	1 (0)	0 (0)	
Fatal		0 (0)	0 (0)	0 (0)	
Access site vascular complication (MVARC)	603				
any		45 (8)	19 (6)	26 (9)	0.107
Minor		44 (7)	18 (6)	26 (9)	
Major		1 (0)	1 (0)	0 (0)	
Pericardiocentesis or emergency surgery	603	5 (1)	1 (0)	4 (1)	0.187
Unplanned surgical intervention because of access site vascular complication	604	9 (2)	7 (2)	2 (1)	0.188
Stroke (in-hospital or < 30 days)	620	8 (1)	4(1)	4 (1)	1.000
Myocardial infarction (in-hospital or < 30 days)	620	0 (0)	0 (0)	0 (0)	1.000

*MV* = mitral valve, *MVARC* = Mitral Valve Academic Research Consortium, *TV* = tricuspid valve

### In-hospital clinical course and complications

Length of post-procedural hospital stay in intensive or intermediate care unit (2.0 ± 2.4 versus 2.6 ± 3.9 days; *p* = 0.003; mean difference 0.6 ± 0.3 days; *p* = 0.027) and total post-procedural hospital stay (6.3 ± 5.1 versus 8.0 ± 7.1; *p* ≤ 0.001; mean difference 1.6 ± 0.5 days; *p* < 0.001) were significantly longer in frail compared to non-frail patients.

**Fig. 1:**
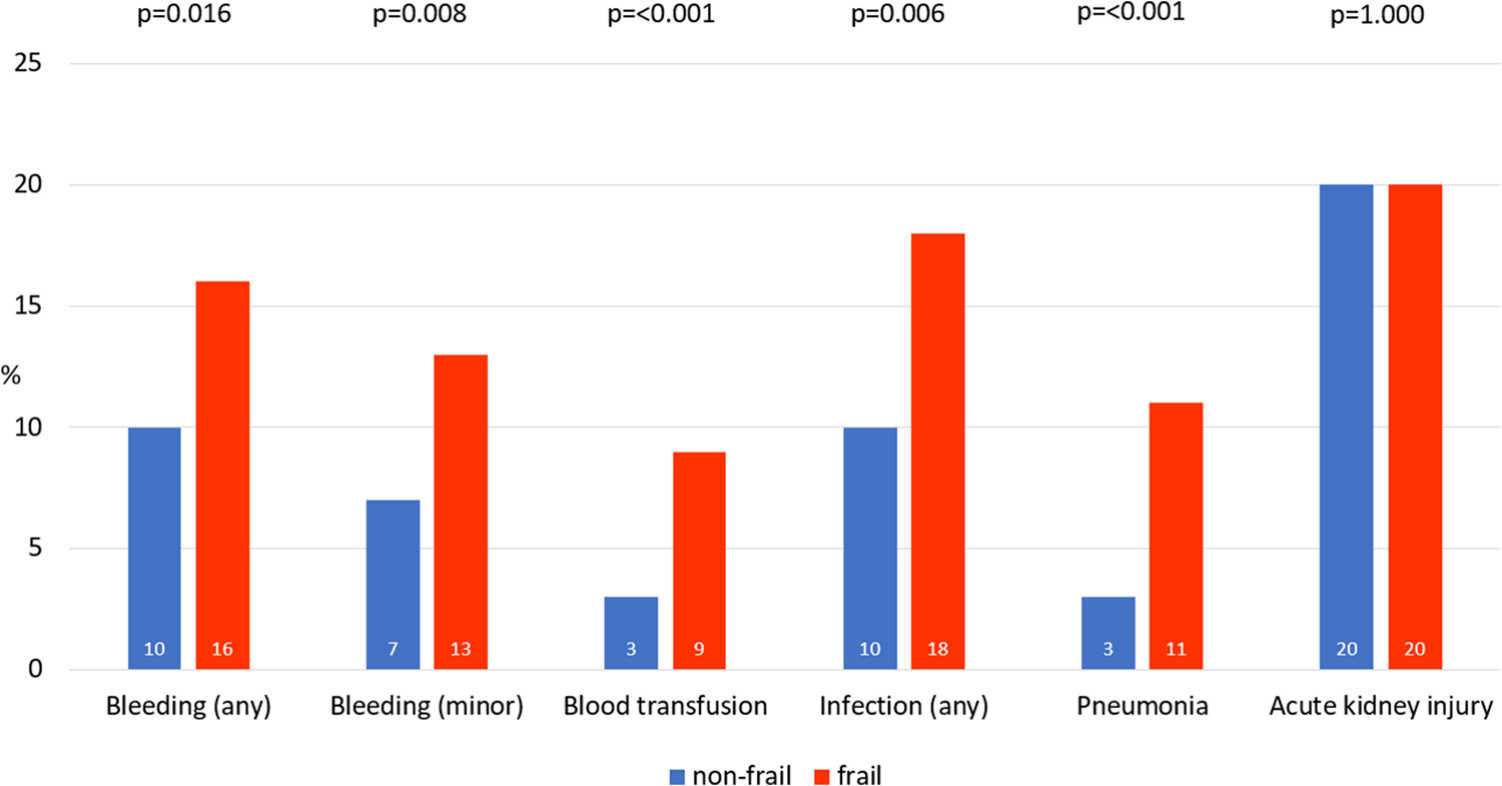
Frequency of periinterventional complications by frailty status. Frequency of periinterventional complications by frailty status in %, *p*-values for difference of incidence between frail and non-frail patients

Frail patients had significantly more bleeding complications in terms of any and minor MVARC bleeding and bleeding requiring blood transfusion (Fig. 1). The most common sites of bleeding were access site (39%), gastrointestinal tract (25%), urological tract (10%) and jugular central venous catheter (10%). The distribution of bleeding locations was similar between groups. Infections, particularly pneumonias, were significantly more common in frail patients (Fig. 1), whereas urinary tract infections were similar between frail and non-frail patients (4.1 vs 3.3%; *p* = 0.623). The incidence of AKI was similar between frail and non-frail patients (Fig. 1).

**Fig. 2:**
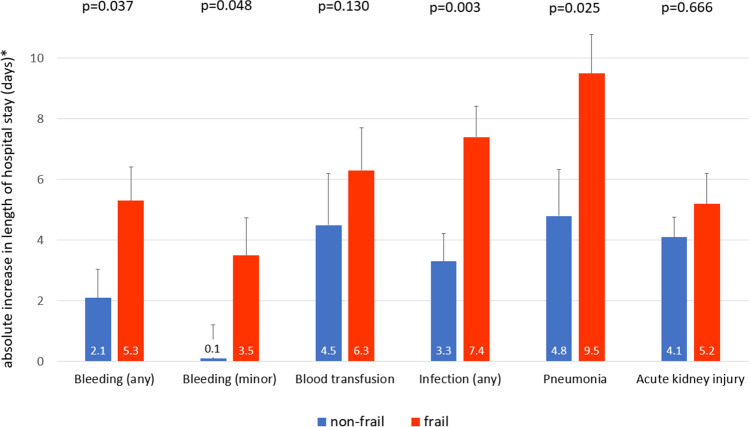
Effect estimate* of periinterventional complications on length of hospital stay by frailty status. *unstandardized regression coefficient from linear regression analysis and standard deviation. *p*-values for interaction by frailty status

The complications AKI, MVARC bleeding, blood transfusions, total infections and pneumonia were significantly associated with length of hospital stay in the overall population (all *p* < 0.001). The increase in length of stay associated with each complication was numerically more pronounced in frail compared to non-frail patients, with a 0.9 to 4.7 day difference in the increase in length of stay after respective complications. The association of MVARC bleeding, total infections and pneumonia with length of hospital stay showed a statistically significant interaction by frailty status (Fig. 2).

Frailty remained significantly associated with length of hospital stay after adjustment for AKI, any MVARC bleeding and total infections, with an attenuated 1.2 ± 0.5 day increase associated with frailty (*p* = 0.010).

### Long-term follow-up

**Fig. 3:**
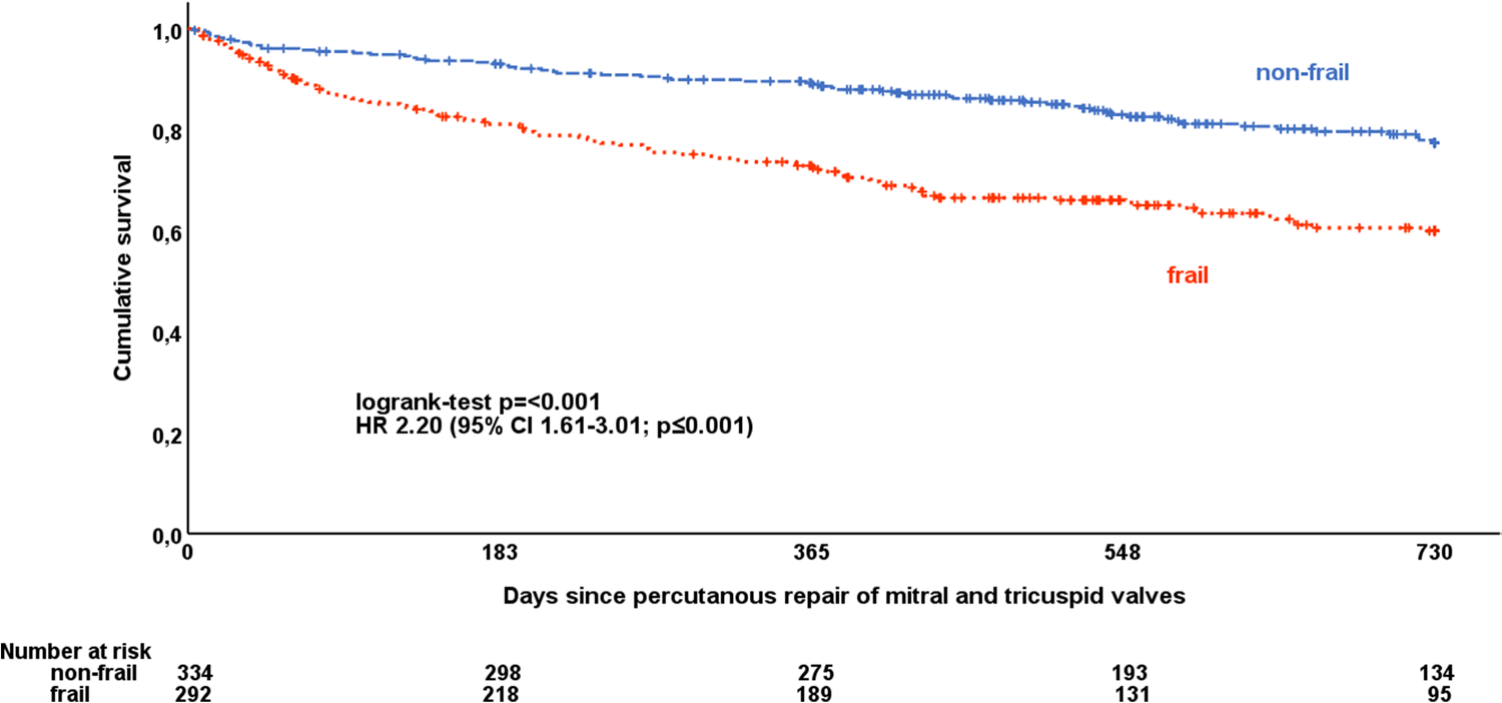
Kaplan-Meier survival plot for all-cause mortality by frailty status. Plot of survival functions for frail patients versus non-frail patients. CI = confidence interval, HR = hazard ratio

The median follow-up time was 560 days (IQR: 363 to 730 days). Frail patients had significantly worse survival compared to non-frail patients (Fig. 3). The estimated 1-year survival rate was 73 ± 3% in frail patients and 89 ± 2% in non-frail patients (log-rank test *p* < 0.001). The estimated 2-year survival rate was 60 ± 3% in frail patients and 77 ± 3% in non-frail patients (log-rank test *p* < 0.001). The hazard ratio (HR) for all-cause death was 2.20 (95% CI 1.61–3.01; *p* < 0.001) for frail patients compared to non-frail-patients.

AKI, blood transfusions, total infections and pneumonia were significantly associated with reduced survival in the overall population, with a similar trend in both frail and non-frail patients (Table 3). The association of total infections and pneumonia with mortality was more pronounced in frail compared to non-frail patients, with no significant interaction.

**Table 3:** Unadjusted hazard of mortality for periinterventional complications in the total population and by frailty status

	Overall (*n* = 626)		Non-frail (*N* = 334)		Frail (*N* = 292)		Inter-action
	HR (95% CI)	*p*-value	HR (95% CI)	*p*-value	HR (95% CI)	*p*-value	*p*-value
Bleeding complication (MVARC)							
Any	1.36 (0.89–2.08)	0.162	1.19 (0.54–2.62)	0.662	1.23 (0.74–2.04)	0.435	0.983
Minor	1.37 (0.86–2.20)	0.184	1.18 (0.47–2.94)	0.726	1.20 (0.69–2.08)	0.511	0.998
Bleeding necessitating blood transfusion	2.28 (1.36–3.82)	0.002	2.03 (0.64–6.46)	0.233	1.79 (1.00–3.21)	0.049	0.863
Infection (any)	2.47 (1.71–3.59)	< 0.001	1.44 (0.68–3.03)	0.339	2.90 (1.87–4.49)	< 0.001	0.103
Pneumonia	3.38 (2.15–5.31)	< 0.001	1.45 (0.45–4.64)	0.529	3.70 (2.19–6.06)	< 0.001	0.141
Acute kidney injury (MVARC)	2.25 (1.61–3.14)	< 0.001	2.38 (1.40–4.05)	0.001	2.35 (1.53–3.61)	< 0.001	0.958

*CI* = confidence interval, *HR* = hazard ratio, *MVARC *= Mitral Valve Academic Research Consortium

Frailty remained significantly associated with mortality after adjustment for AKI, blood transfusions and total infections (HR 2.24 [95% CI 1.62–3.10]; *p* < 0.001).

Subgroup analyses of patients undergoing MV repair with MitraClip (*n* = 484) and patients undergoing other procedures (*n* = 123) showed similar differences by frailty status with respect to frequency of bleeding, infection and AKI, associated increase in length of hospital stay and the hazard ratio of all-cause mortality (Supplementary Fig. 1a and 1b).

## Discussion

This is the first study to investigate the frequency and prognostic impact of periinterventional clinical complications by frailty status in patients undergoing TMTVR. The main finding is that bleeding complications and infections were significantly more common in frail patients whereas the frequency of AKI was similar in frail and non-frail patients. Infection, pneumonia, bleeding complications and AKI were significantly associated with longer hospital stay, which was more pronounced in frail patients. The strong association between frailty and long-term mortality was not mediated by periprocedural complications.

Current guidelines recommend that all patients with valvular heart disease should be assessed for frailty during the treatment evaluation process [1], and the frequency of frailty is particularly high in patients treated by percutaneous interventions. However, there is a lack of data on the impact of frailty on the acute and mid-term outcomes after TMTVR. Infections were approximately twice as common in frail patients as in non-frail patients. The accumulation of infections in frail patients was driven by pneumonias. General anaesthesia with endotracheal intubation was performed in all our patients and is a general risk factor for the development of pneumonias [14]. Frailty in the general population is associated with increased susceptibility and severity of pneumonia in older adults suggesting a particular vulnerability of frail people to this disease condition. One underlying mechanism may be sarcopenic dysphagia in frail patients, which may favour micro-aspiration of oropharyngeal colonising germs and subsequent development of aspiration pneumonia [15]. Reduced cough reflex and respiratory muscle strength, which may be more common in frail adults due to sarcopenia or cerebrovascular disease, may also be an underlying mechanism [16].

Bleeding was also more frequent in frail patients than in non-frail patients. The higher rate of bleeding may be explained by poorer renal function, as chronic kidney disease is a general risk factor for major bleeding in patients with cardiovascular disease [17]. Possible mechanisms include platelet dysfunction and interactions with antiplatelet therapy and anticoagulation [18, 19]. As atrial fibrillation is significantly more common in frail patients, a higher proportion of oral anticoagulation and bridging strategies may explain the increased risk of bleeding. Also, higher rates of postoperative delirium and agitation in frail patients may lead to increased self-mobilisation early after the intervention and subsequently increased postoperative access site related bleeding. Finally, the lower baseline haemoglobin levels in frail patients may lead to an increase likelihood of blood transfusions even after mild blood loss.

There was no difference in the incidence of AKI according to frailty status. This finding is consistent with evidence in patients undergoing transfemoral aortic valve replacement[20] and suggests that the impact of frailty on AKI after valve interventions is likely to be small compared to other known risk factors such as heart failure, diuretic usage, urgent procedure and device failure [21, 22].

The increased incidence of certain complications in frail patients is of major clinical and economic relevance given the association with longer hospital stays. We have shown earlier that frailty is associated with a mean 32% increase in total hospital costs after MitraClip procedure driven by longer stay on intensive care unit and regular ward [23]. We now extend these findings by demonstrating that the prolonged length of stay in frail patients is at least partly explained by more common periprocedural complications and a stronger clinical impact of these complications. Our findings support the understanding of frailty as a state of increased vulnerability to stressors leading to an increased risk of adverse health outcomes. We extend existing evidence from other clinical settings such as cancer and hip fracture patients, where frailty is associated with higher rates of complications such as AKI, delirium and pneumonia, as well as longer hospital stays, higher mortality and hospital costs [24, 25]. Firstly, the TMTVR procedure including general anaesthesia acts as an external stressor causing more complications such as infections or bleeding in frail patients. Secondly, periprocedural complications such as infections act as internal stressors that have a greater impact in frail patients with significantly longer hospital stay due to slower recovery. Importantly though, frailty was an independent predictor of longer hospital stay after adjustment for periprocedural complications. Possible explanations may be other frailty characteristics such as multimorbidity including heart failure, atrial fibrillation, or chronic kidney disease which can contribute to longer hospital stay via delayed physical mobilization or the need of adapting medical therapies [26–28].

The occurrence of postprocedural complications is of great clinical relevance due to their association with higher mortality. Blood transfusion and AKI are well-established risk factors in Mitraclip patients [29, 30]. However, this is the first study to show a significant association of infections, particularly pneumonia, with higher mortality after TMTVR. It is beyond the scope of this study to prove a causal relationship between postprocedural complications and mortality, as the cause of death was not available for most patients. However, the association between individual complications and death was statistically significant already after 6 weeks of follow-up (data not shown), which may indicate at least a partial causal contribution. Although the prognostic impact of periprocedural complications was largely independent of whether patients were frail or not, it is particularly important in the management of frail patients who have an almost doubled risk of postprocedural complications.

As a result, frail patients may particularly benefit from prevention of these complications or extra vigilance after TMTVR to detect and treat complications earlier. For example, the use of conscious sedation or deep sedation instead of general anaesthesia may help to reduce the risk of pneumonia [31]. Other preventive measures might include treatment of sarcopenic dysphagia to reduce aspiration pneumonia through resistance training of the swallowing muscles, and nutritional support which could support the concept of pre-rehabilitation [32, 33]. Bleeding complications and blood transfusions could be reduced by avoiding jugular central venous catheters, preoperative patient blood management including treatment of iron deficiency anaemia which is common in TMTVR patients and judicious perioperative use of antiplatelet therapy and anticoagulants [34, 35].

### Study limitations

Due to the retrospective nature of our study, the results may be susceptible to confounding or information bias due to incomplete medical records. For example, incomplete documentation of clinical complications may have led to an underestimation of the frequency estimates. However, compared with other studies of patients undergoing MitraClip, the absolute incidence of perioperative complications in our study is similar [22, 36, 37]. Furthermore, this may not affect our conclusions because the physicians were blinded to the frailty status of the patients and therefore under-reporting may be independent of frailty status. As discussed, the observational design does not allow conclusions to be drawn about the causality of the association between frailty and complications and between complications and subsequent outcomes. Furthermore, as this was a single-centre study, our results regarding procedural success and periprocedural complications need to be interpreted in the context of a single-centre team of experienced operators. While a subgroup analysis confirmed consistent results in MV Clip and non-MV Clip patients, the generalisability of our results to each individual non-MV Clip procedure is limited due to the small number of patients undergoing each individual valve repair technique.

Finally, the absolute length of stay after TMTVR varies between health care systems and may be longer in Germany than, for example, in the United States. Nevertheless, the relative increases associated with frailty and complications may be applicable to other systems.

## Conclusion

Bleeding complications, blood transfusion and infections were almost twice as common in frail compared to non-frail patients undergoing TMTVR and partly explained the prolonged hospital stay in frail patients. Further considering the association of these complications with subsequent mortality, particularly frail patients may benefit from extra vigilance after TMTVR, and future effort should be spent on preventing these complications. The strong association between frailty and long-term mortality was not mediated by periprocedural complications.

## Supplementary information

Below is the link to the electronic supplementary material.

Supplementary file1 (DOCX 184 KB)

## Data Availability

The data underlying this article are available in the article.
