# Supplementary material for: Frailty, periinterventional complications and outcome in patients undergoing percutaneous mitral and tricuspid valve repair
Source: Clin Res Cardiol. 2024 Feb 15;114(7):827–35. doi: 10.1007/s00392-024-02397-3 (PMC12202524; doi:10.1007/s00392-024-02397-3)
Supplement: Supplementary file 1 — Supplementary file1 (DOCX 184 KB) [file 392_2024_2397_MOESM1_ESM.docx]

**Supplementary Figure 1a: Subgroup analysis for patients undergoing MV repair with MitraClip (n=484)**


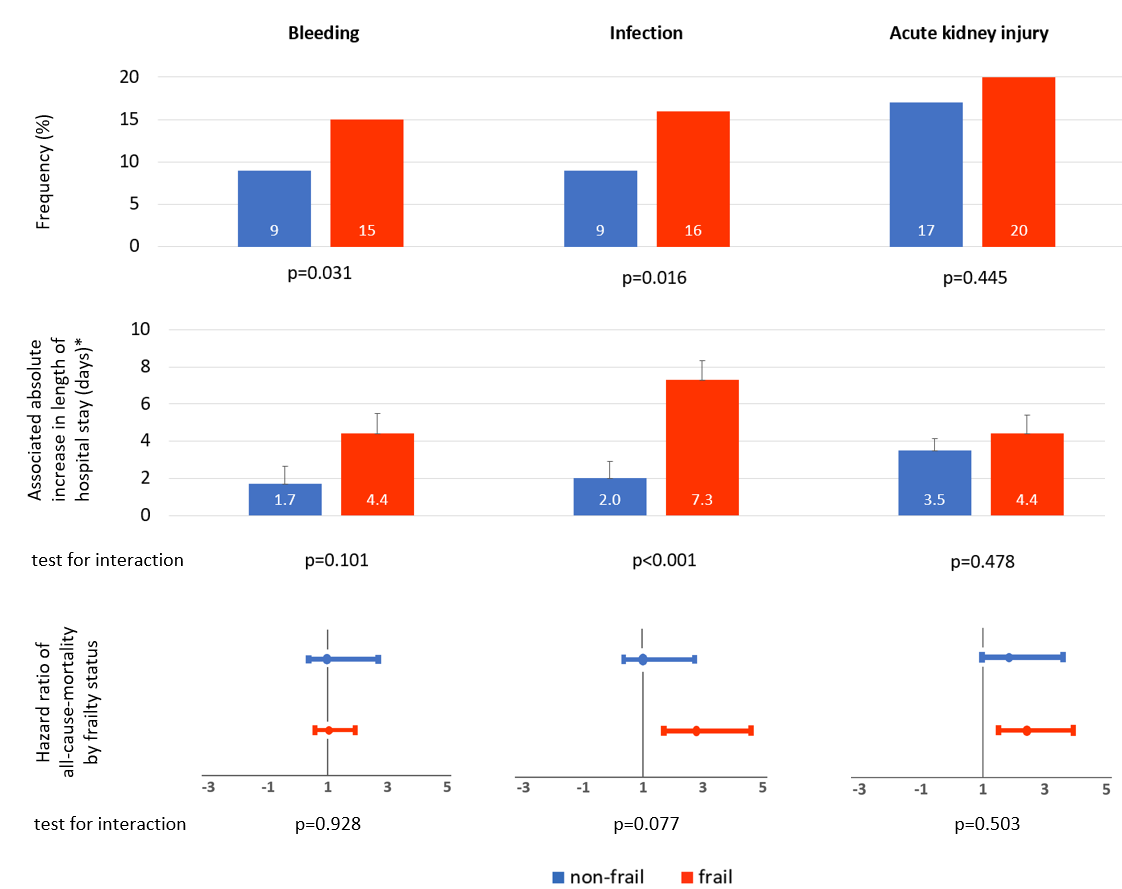


*unstandardized regression coefficient from linear regression analysis and standard deviation

p-values for interaction by frailty status

**Supplementary Figure 1b: Combined subgroup analysis for all other procedures except MV repair with MitraClip (n=123)**


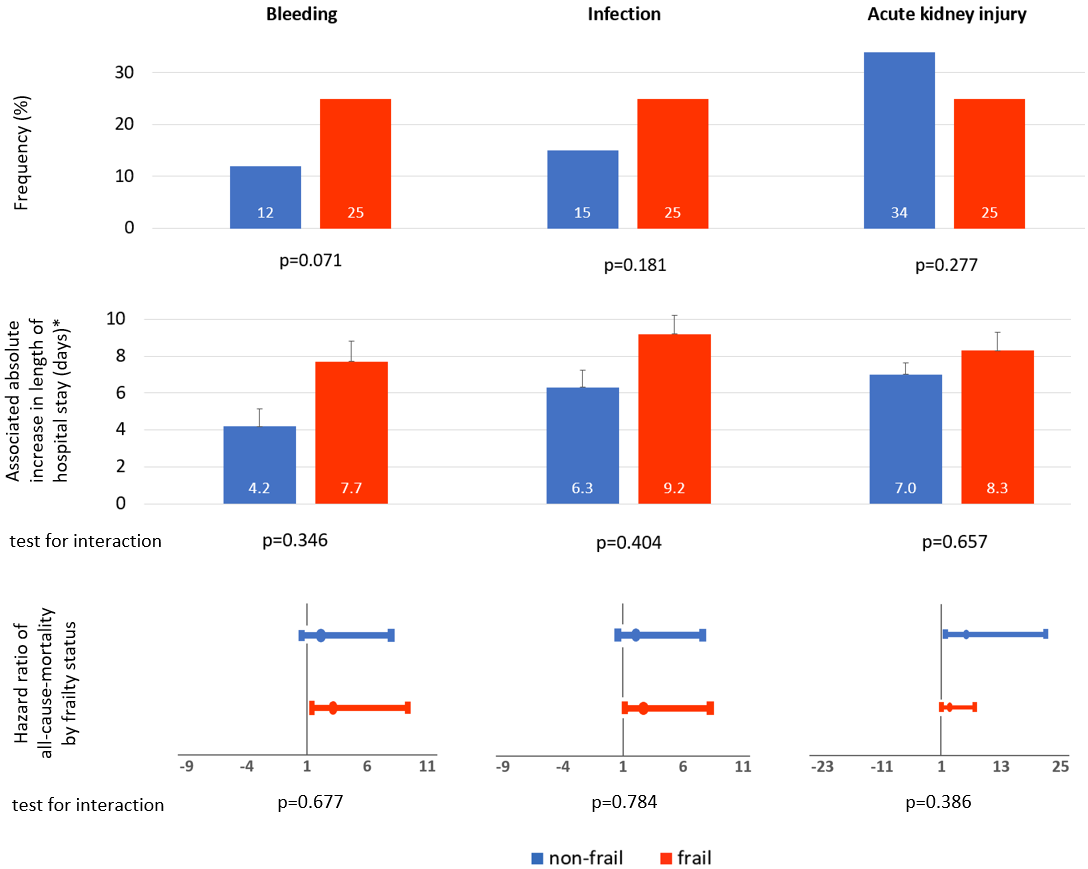


*unstandardized regression coefficient from linear regression analysis and standard deviation

p-values for interaction by frailty status
